# Prevalence and Associated Factors of Chronic Fatigue Among Healthcare Professionals: A Cross-Sectional Study at a University Hospital in North Africa

**DOI:** 10.3390/healthcare13243245

**Published:** 2025-12-11

**Authors:** Rim Ghammam, Ismail Dergaa, Sihem Ben Fredj, Nawel Zammit, Rim Akrimi, Halil İbrahim Ceylan, Valentina Stefanica, Bassem Charfeddine, Imed Harrabi, Jihene Maatoug, Houda Kalboussi

**Affiliations:** 1 Department of Preventive and Community Medicine, Farhat Hached University Hospital, Sousse 4000, Tunisia; ghammam.rim2013@gmail.com (R.G.); sihembenfredj2015@gmail.com (S.B.F.); nawel.zommit@gmail.com (N.Z.); imed.harrabi@yahoo.com (I.H.); jihenmaatoug3107@gmail.com (J.M.); 2 Doctoral School, Faculty of Medicine of Sousse, University of Sousse, Sousse 4002, Tunisia; 3 Higher Institute of Sport and Physical Education of Ksar Said, University of Manouba, Manouba 2010, Tunisia; phd.dergaa@gmail.com; 4 Physical Activity, Sport and Health Research Unit, National Observatory of Sports, Tunis 1003, Tunisia; 5 Faculty of Medicine of Sousse, University of Sousse, Sousse 4002, Tunisia; akrimirim9@gmail.com (R.A.); charfeddinebassem@gmail.com (B.C.); drhoudakalboussi@hotmail.fr (H.K.); 6 Physical Education and Sports Teaching Department, Faculty of Sports Sciences, Atatürk University, 25240 Erzurum, Türkiye; 7 Department of Physical Education and Sport, Faculty of Sciences, Physical Education and Informatics, National University of Science and Technology Politehnica Bucharest, Pitești University Center, 060042 Pitești, Romania; 8 Department of Biochemistry, Farhat Hached University Hospital, Sousse 4002, Tunisia; 9 Department of Occupational Medicine, Farhat Hached University Hospital, Sousse 4002, Tunisia

**Keywords:** chronic fatigue, chronic fatigue syndrome, depression, healthcare workers, North Africa, obesity, occupational health, quality of life, Tunisia

## Abstract

Background: Chronic fatigue constitutes a critical occupational health challenge among healthcare workers with substantial implications for individual well-being and patient safety. Empirical evidence regarding chronic fatigue among healthcare professionals in North Africa remains limited, particularly in contexts where healthcare systems contend with resource constraints and elevated workload demands. Objective: This study aimed to determine the prevalence of chronic fatigue and chronic fatigue syndrome (myalgic encephalomyelitis (CFS/ME)) among healthcare professionals without severe chronic diseases at a university hospital in Tunisia and identify independent associations of chronic fatigue. Methods: We conducted a cross-sectional analysis of 205 healthcare professionals at University Hospital Farhat Hached, Sousse, Tunisia, from October to December 2021. Data were collected using a pre-test questionnaire to collect data about sociodemographic characteristics, chronic fatigue, quality of life, and lifestyle habits. A vitamin D3 test was also performed, as it is identified as an associated factor and potential biological modulator of chronic fatigue. Results: Chronic fatigue prevalence was 37.1%, with chronic fatigue syndrome (myalgic encephalomyelitis (CFS/ME)) prevalence of 11.2%. Multivariate analysis revealed good physical health-related quality of life (adjusted Odds Ratio (OR) = 0.08, *p* < 0.001) and good mental health-related quality of life (adjusted OR = 0.10, *p* < 0.001) as protective factors. Moderate-to-severe depression (adjusted OR = 5.84, *p* < 0.001) and obesity (adjusted OR = 2.50, *p* = 0.021) independently increased chronic fatigue risk. No independent association was detected between vitamin D levels and chronic fatigue. Conclusions: Chronic fatigue affects more than one-third of healthcare professionals in this resource-limited setting. Comprehensive occupational health interventions addressing psychological and metabolic health factors are needed to protect healthcare workers’ well-being and maintain the quality of care delivery.

## 1. Introduction

Fatigue is a prevalent complaint in clinical practice and the general population, ranging from transient tiredness to persistent states that interfere with daily activities and occupational functioning [1].

Chronic fatigue is persistent fatigue, often linked to identifiable causes (illness, stress, sleep disorders, etc.). It can last for several weeks or months, but is not associated with specific symptoms or significant disability [2,3]. There is no standardized definition, and diagnosis is based on identifying and treating the cause. It usually disappears when the underlying cause is treated [2,3].

Chronic fatigue syndrome is also known as myalgic encephalomyelitis (CFS/ME) [4]. It constitutes a significant global public health challenge [5]. Meta-analyses and systematic reviews indicated a global prevalence of SFC/ME ranging from 0.2% to 1.4% [5,6]. Using CDC-1994 criteria, the prevalence is generally observed to range from 0.65 to 0.89% [5]. The prevalence is consistently higher in females (1.36%) than in males (0.86%), with a female-to-male ratio of 1.5–2.1 [5,6]. In the United States, a national survey reported a prevalence of 1.2% to 1.3% [6,7]. In Europe, prevalence ranges from 0.1% to 2.2% [8]. Its prevalence varies depending on the diagnostic criteria, and its origin remains poorly understood [5].

CFS/ME manifests as unexplained chronic fatigue lasting more than 6 months, not relieved by rest, associated with post-exertional malaise, sleep disturbances, muscle/joint pain, cognitive impairment, and other multisystemic symptoms [4,6]. The absence of specific biomarkers makes diagnosis difficult, relying on exclusion of different pathologies and on clinical criteria (Fukuda, CDC, Canada, SEID) [4,6]. The impact on quality of life is significant, with high rates of disability and unemployment. The disease burden manifests through severe long-term disability, reduced quality of life, and increased healthcare utilization [5,9].

Although etiological mechanisms remain incompletely elucidated, evidence supports multifactorial pathophysiological models incorporating neuroimmune dysfunction, mitochondrial abnormalities, and metabolic perturbations [1].

Fatigue has been correlated to many factors, including sociodemographic characteristics, occupational exposure, lifestyle habits, and biological factors. Depression and anxiety represent prevalent comorbidities with likely bidirectional relationships to fatigue [9,10]. Vitamin D has pleiotropic roles in musculoskeletal health, immune regulation, and brain function. Many studies reported lower 25-hydroxyvitamin D (25OHD) levels among patients with chronic fatigue. They reported a plausible link between vitamin D deficiency and fatigue via immune and neuroendocrine mechanisms, though definitive causal relationships remain unestablished [11,12].

Healthcare professionals face multiple risk factors for chronic fatigue development, including substantial workloads, irregular scheduling, night shifts, occupational stress, and infectious disease exposure [13,14]. Chronic fatigue prevalence among healthcare workers reaches concerning levels, with studies reporting up to 84% experiencing significant fatigue [13,15]. Fatigue is associated with reduced health status, diminished quality of life, and, critically, increased medical error risk directly compromising patient safety [15,16]. During the SARS-CoV-2 pandemic, elevated prevalence rates of fatigue, burnout, and mental health symptoms were documented among healthcare providers globally [17].

Several studies have reported the prevalence of chronic fatigue. However, in Tunisia and comparable low- and middle-income countries (LMICs), there is limited empirical evidence on chronic fatigue among healthcare populations [18]. To our knowledge, no study has reported this problem in our context, especially among the health population. Published data examining chronic fatigue among healthcare workers without severe chronic comorbidities remain scarce in North African contexts [19]. 

Since the Arab Spring, Tunisia has been experiencing political, social, and economic crises. The COVID-19 pandemic has exacerbated this situation. Third-line hospitals, such as Farhat Hached Hospital, were not spared, facing a growing shortage of human and material resources with increasing workload. This study aimed to (i) determine the prevalence of chronic fatigue and chronic fatigue syndrome (myalgic encephalomyelitis (CFS/ME)) among healthcare professionals without severe chronic diseases at a tertiary university hospital in Tunisia, and (ii) examine associations between chronic fatigue and sociodemographic characteristics, occupational factors, health-related quality of life, depressive symptoms, and serum vitamin D levels.

## 2. Materials and Methods

### 2.1. Ethical Approval

The study protocol received approval from the Ethics Committee of the Faculty of Medicine Ibn Al-Jazzar (reference number: CEFMSo_006725). Free, informed, written consent was obtained from each participant before enrollment. The research adhered to the principles of the Declaration of Helsinki. Data were collected by the project medical team and treated anonymously. Participants received laboratory results and were referred to occupational health services when clinically indicated.

### 2.2. Study Design and Setting

This cross-sectional study reports baseline assessment data from an intervention trial investigating chronic fatigue, quality of life, and depression before and after vitamin D3 supplementation among healthcare workers. The study was conducted at University Hospital Farhat Hached, Sousse, Tunisia, from October to December 2021. The hospital represents one of Tunisia’s leading tertiary university medical centers, providing specialized care for the central-eastern region and serving as a referral center for complex conditions. It is a teaching hospital, classified as a Category “A” hospital and considered an essential medical center that offers a wide range of medical specialties, high-level therapeutic services, and is actively involved in medical education and healthcare research. In 2020, it received the first case of COVID-19 infection in Tunisia.

The hospital is a large facility that can house up to 705 beds and provide approximately 42 medical and medical–technical services, including many specialties and departments. It employed a diverse staff of over 1900 healthcare professionals, comprising physicians, nurses, care assistants, and administrative staff.

### 2.3. Sample Size and Sampling

Sample size was calculated based on type I error α = 5%, precision of 5%, and an estimated chronic fatigue prevalence of 14.6% [20,21]. The required sample size was 192 participants; ultimately, 205 healthcare professionals were enrolled. Participants were randomly selected from all healthcare departments.

**Figure 1:**
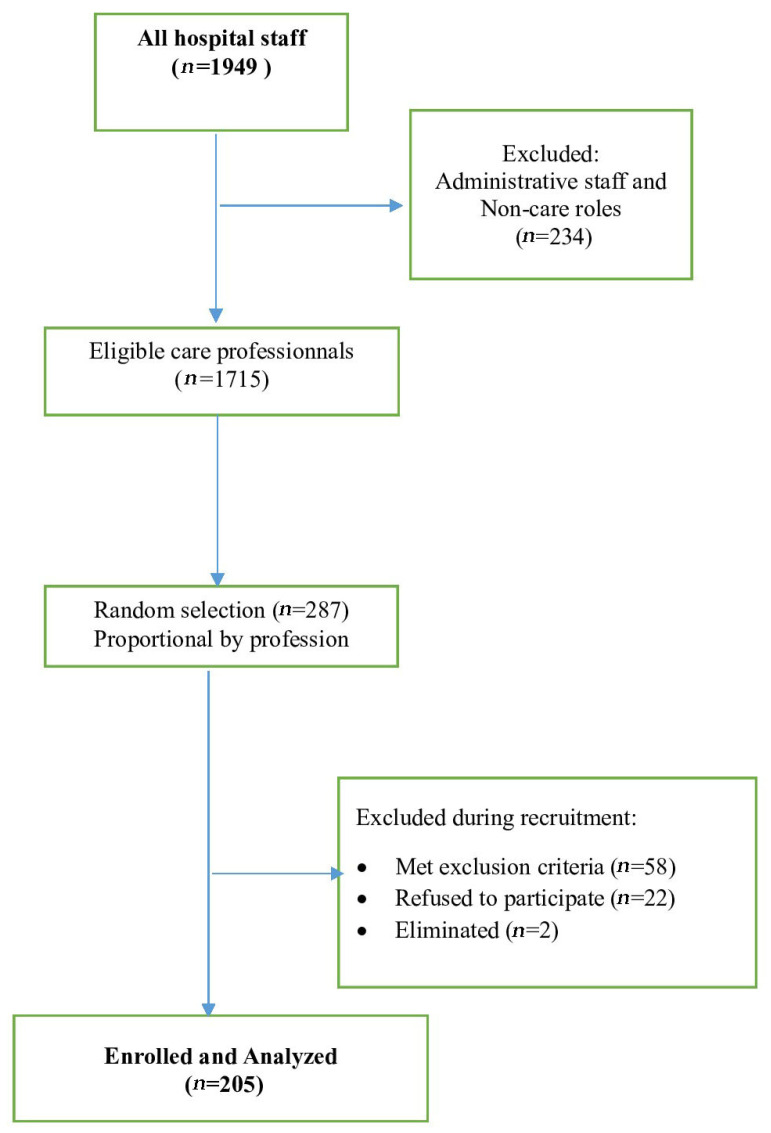
Flowchart of participant recruitment among healthcare professionals at Farhat Hached University Hospital, Sousse, Tunisia (October–December 2021).

First, an exhaustive list of all permanent professional staff was obtained from the hospital administration, totaling 1949 professionals. We systematically excluded administrative staff and non-care professionals (*n* = 234), leaving a core population of 1715 healthcare professionals eligible for care activity. Second, we calculated the proportion of physicians among healthcare professionals (18%, 309/1715) and ensured the initial random selection pool was proportionally representative of the professional categories. Finally, we used a simple random sampling technique applied to the entire eligible list (*n* = 1695). This was done using Excel’s RANDOM function to ensure every eligible professional had an equal probability of selection. During recruitment, 58 participants met exclusion criteria, including 20 professionals with imminent retirement and 30 who fulfilled other predefined criteria. Of those contacted, 22 individuals declined participation, yielding an acceptance rate of 90.3% (205/227). Following inclusion, two participants were subsequently excluded due to severe anemia and uncontrolled hypertension. Ultimately, 205 participants were enrolled and included in the final analysis (Figure 1).

Inclusion criteria: Healthcare workers employed at the hospital for at least 12 months who signed informed consent.

Exclusion criteria: Subjects with conditions potentially causing severe fatigue or vitamin D deficiency [12] including vitamin D supplementation in the preceding 8 weeks; high-dose calcium supplements (>1000 mg daily); pregnancy or breastfeeding; contraindications to vitamin D supplementation; uncontrolled endocrine disorders; advanced or active malignancy; severe anemia (hemoglobin <9.0 g/dL); advanced neurological, cardiac, pulmonary, renal, or hepatic disease; advanced rheumatologic disorders; established sleep disorders; known psychiatric disorders; conditions altering vitamin D metabolism; planned retirement within 12 months; and administrative staff positions.

Elimination criteria: Severe conditions revealed by biological analysis or clinical examination following enrollment.

### 2.4. Measurements

#### 2.4.1. Sociodemographic and Clinical Data

A pre-test questionnaire in French collected data on sociodemographic characteristics, medical history (we collected information regarding the medical and surgical history of healthcare professionals (yes/no question), as well as medication use and the severity of their condition), working characteristics (seniority in the profession (number of years and months in the position and profession, night work (yes/no), number of on-call shifts per month), etc.). Dietary habits and sun exposure were also collected using a 4-point Likert scale. 

Biometric measurements included weight (nearest 0.1 kg, portable electronic scale), height (standing position, bare feet, nearest 0.5 cm), and waist circumference. Blood pressure was measured twice at rest using an arm electronic sphygmomanometer, and the mean values were calculated.

Body mass index was computed as weight/height^2^ (kg/m^2^). Overweight and obesity were defined using World Health Organization criteria [22].

#### 2.4.2. Chronic Fatigue Using Multidimensional Fatigue Inventory (MFI-20)

The MFI-20 [23] is a 20-item self-report questionnaire measuring fatigue across five dimensions: general fatigue (items 1, 5, 12, and 16), physical fatigue (items 2, 8, 14, and 20), reduced activity (items 7, 11, 13, and 19), reduced motivation (items 3, 6, 10, and 17), and mental fatigue (items 4, 9, 15, and 18). The instrument demonstrates good internal consistency (Cronbach’s α = 0.84). [24]. Responses are recorded on a five-point Likert scale (1 = “yes, that is true” to 5 = “no, that is not true”), with higher scores indicating greater fatigue. Positively worded items require reverse scoring (items 2, 5, 9, 10, 13, 14, 16, 17, 18, and 19). Each dimension score ranges from 4 to 20 points. The overall fatigue score represents the sum of all 20 items. A total score ≥43.5 indicates moderate-to-severe fatigue (chronic fatigue “yes”) [24]. Subscale cutoffs are ≥11 for general fatigue, ≥10 for reduced activity, and ≥9 for other subscales [24].

#### 2.4.3. Chronic Fatigue Syndrome (Myalgic Encephalomyelitis (CFS/ME)) Using the Chronic Fatigue Syndrome Severity Scale

The CFS Severity Scale [25] is a self-administered questionnaire in which subjects rate the intensity of fatigue and eight symptoms during the preceding six months on a five-point scale (0 = no symptoms to 4 = severe symptoms). Total score ranges from 0 to 32. The tool demonstrates good internal consistency (Cronbach’s α = 0.924) [25]. The total score is the sum of the values of 8 items, which range from 0 to 32. A score <14 with no to mild fatigue indicates no chronic fatigue. A score <14 with moderate-to-severe fatigue indicates idiopathic chronic fatigue. A score ≥14 with no or mild fatigue indicates a chronic fatigue-like syndrome without severe fatigue. Chronic fatigue syndrome (“yes”) (CFS/ME) is present when we have a score that indicates an idiopathic chronic fatigue [25].

#### 2.4.4. Health-Related Quality of Life Using the SF-12 Health Survey

The SF-12 questionnaire [26,27] provides a reliable measurement of health-related quality of life through 12 items across eight domains, yielding Physical Component Summary (PCS-12) and Mental Component Summary (MCS-12) scores. The French version shows satisfactory reliability (Cronbach’s α > 0.70) [27]. Scores of MCS-12 or PCS-12 ≥ 50 indicate good mental and physical health-related quality of life.

#### 2.4.5. Depression Using Patient Health Questionnaire-9 (PHQ-9)

The PHQ-9 [28] is a self-reported screening tool for depression with excellent psychometric properties (Cronbach’s α > 0.86 in English and >0.81 in French) [28,29]. The scale comprises 10 items that explore symptoms over the preceding 2 weeks. The maximum score is 27 points. Depression (according to the PHQ-9 questionnaire): Items 1–9 are rated on a scale from 0 (not at all) to 3 (nearly every day). Item 10 (level of functioning) is rated on a scale of 0 to 4, ranging from “not at all difficult” to “extremely difficult.” Depression severity thresholds are no depression (0–4), mild (5–9), moderate (10–14), moderately severe (15–19), and severe depression (20–27).

### 2.5. Laboratory Measurements

Lipid and phosphocalcic assessments were conducted for all participants using DX Technolab (BECKMAN). Vitamin D3 measurement was performed in the hospital biochemistry laboratory using standardized assay techniques. The machine used for vitamin D3 testing was the COBAS E411 analyzer (HİTATHİ). ROCHE manufactured the kits.

Vitamin D status was classified according to established thresholds [30]: normal (25-hydroxyvitamin D ≥ 30 ng/mL), insufficiency (21–29 ng/mL), and deficiency (≤20 ng/mL).

### 2.6. Statistical Analysis

Quantitative variables are described using means and standard deviations, with medians and interquartile ranges (IQRs) for non-normally distributed variables. Qualitative variables are described using frequencies and percentages. Bivariate associations were examined using chi-square tests or Fisher’s exact test, where appropriate. Multivariable binary logistic regression identified factors independently associated with chronic fatigue, with adjusted odds ratios and 95% confidence intervals reported. Variables with *p* < 0.2 in bivariate analyses or clinical relevance were considered for multivariable models. Variables showing strong multicollinearity with the dependent variable were excluded to maintain the stability and reliability of the regression model. Statistical significance was set at α = 0.05. SPSS version 25.0 was used for analysis.

## 3. Results

### 3.1. Participant Characteristics

Figure 1 illustrates the study’s flowchart. The acceptance rate was 90.31% (205/227).

Table 1 presents characteristics of the 205 healthcare professionals. The population was predominantly female (89.3%, *n* = 183), with a male-to-female ratio of 0.12. Mean age was 43.55 ± 8.32 years. Age distribution showed 46.8% (*n* = 96) aged ≤40 years. Regarding occupation, nurses accounted for 43.9% (*n* = 90), health technicians for 38.0% (*n* = 78), and physicians for 18.0% (*n* = 37). Night work was reported by 27.8% (*n* = 55).

### 3.2. Chronic Fatigue and Chronic Fatigue Syndrome (Myalgic Encephalomyelitis (CFS/ME)) Prevalence

Data presented in Table 2 suggest that chronic fatigue is highly prevalent in the study population, affecting 37.1% participants. The overall chronic fatigue score was 54.5 ± 14.32, with a median of 55 and an interquartile range (IQR) of 43–65. Fatigue experienced by our population is dominated by physical fatigue (78.5%). General fatigue showed a mean score of 12.40 ± 3.95, with 66.3% (*n* = 136) meeting threshold criteria. Mental fatigue had the lowest mean score (9.59 ± 2.57) and was present in 67.3% (*n* = 138). 

Table 3 presents CFS prevalence. Mean CFS score was 8.24 ± 5.14 (median 7, IQR 4–12, range 0–23). Idiopathic chronic syndrome was present in 22.0% (*n* = 45), CFS-like syndrome with insufficient fatigue in 4.4% (*n* = 9), and chronic fatigue syndrome in 11.2% (*n* = 23).

### 3.3. Associated Factors to Chronic Fatigue

Table 4 examines factors associated with chronic fatigue dimensions (according to the MFI-20 scale). Women exhibited significantly higher rates of general fatigue (70.5% vs. 31.8%, *p* < 0.001), reduced motivation (59.0% vs. 31.8%, *p* = 0.015), and total fatigue (39.9% vs. 13.6%, *p* = 0.016) compared to men. Participants aged ≤40 years demonstrated higher rates of general fatigue (74.0% vs. 59.6%, *p* = 0.030) and reduced activity (63.5% vs. 49.5%, *p* = 0.044). Married individuals reported higher physical fatigue rates (81.1% vs. 55.0%, *p* = 0.007).

Regarding vitamin D status, the proportion with total fatigue was 22.6% among participants with deficiency (<20 ng/mL), compared with 42.4% among those with insufficient or normal levels (*p* = 0.011). Poor physical health-related quality of life (PCS-12 < 50) was associated with higher rates across all dimensions, including total fatigue (49.3% vs. 6.8%, *p* < 0.001). Poor mental health-related quality of life (MCS-12 < 50) was associated with all dimensions, including total fatigue (44.2% vs. 7.5%, *p* < 0.001).

Healthcare workers with moderate-to-severe depression exhibited elevated rates of all dimensions of fatigue (obesity associated with higher total fatigue prevalence (53.1% vs. 30.7%, *p* = 0.002). (Table 4).

### 3.4. Independent Predictors of Chronic Fatigue

Table 5 presents multivariable logistic regression results. Good physical health-related quality of life (PCS-12 ≥ 50) was associated with reduced chronic fatigue risk (adjusted OR = 0.08, 95% CI: 0.03–0.25, *p* < 0.001). Good mental health-related quality of life (MCS-12 ≥ 50) was associated with a protective effect (adjusted OR = 0.10, 95% CI: 0.03–0.39, *p* < 0.001). Moderate-to-severe depression increased chronic fatigue risk nearly sixfold (adjusted OR = 5.84, 95%CI: 2.32–14.71, *p* < 0.001). Obesity increased risk 2.5-fold (adjusted OR = 2.50, 95%CI: 1.15–5.46, *p* = 0.021).

## 4. Discussion

### 4.1. Principal Findings

This investigation among 205 healthcare professionals in Tunisia revealed chronic fatigue prevalence of 37.1% and chronic fatigue syndrome prevalence (myalgic encephalomyelitis (CFS/ME)) of 11.2%. General fatigue (66.3%) and mental fatigue (67.3%) demonstrated particularly high prevalence. Multivariate analysis identified depression (sixfold increased risk) and obesity (2.5-fold increased risk) as independent risk factors, while good physical and mental quality of life demonstrated protective effects (approximately 90% risk reduction). No independent association was detected between vitamin D status and chronic fatigue.

### 4.2. Comparison with Existing Evidence

The observed chronic fatigue prevalence substantially exceeds general population estimates of 10–15% for fatigue persisting beyond six months [5,21]. The chronic fatigue syndrome prevalence of 11.2% markedly exceeds the global pooled prevalence estimate of 0.68% (95%CI: 0.48–0.97%) from population-based studies [5], underscoring healthcare workers’ occupational vulnerability. Studies from China reported variable fatigue prevalence, with one recent multicenter study reporting 53.9% among doctors and nurses [17]. Research from high-income countries documented rates reaching 84% among specific subgroups [13]. Healthcare workers in low-and middle-income countries may face additional stressors, including resource constraints, higher patient-to-staff ratios, and limited occupational health support [18].

The elevated rates of general and mental fatigue warrant attention. Mental fatigue encompasses cognitive aspects, including difficulties with concentration [22], and carries particular significance given its associations with impaired clinical decision-making, reduced diagnostic accuracy, and increased error susceptibility [16], directly threatening patient safety.

### 4.3. Demographic Patterns

The pronounced gender difference, with women exhibiting higher chronic fatigue rates across multiple dimensions, must be interpreted cautiously given the sample composition (89.3% female, *n* = 183 vs. 10.7% male, *n* = 22). This gender imbalance limits statistical power for gender comparisons and represents a significant study limitation. While biological factors, including sex hormones and immune regulation differences, may contribute to differential fatigue vulnerability [5,31], sociocultural factors, including domestic workload distribution and childcare responsibilities, may impose a cumulative burden on female healthcare workers [31,32]. The small male sample size necessitates cautious interpretation of gender-related findings.

The age-related pattern, with younger workers (≤40 years) reporting higher general fatigue and reduced activity, aligns with the occupational health literature documenting elevated stress among early-career professionals [10]. Younger workers typically possess less professional experience and may demonstrate incomplete adaptation to demanding conditions [10]. Organizational practices often assign heavier workloads to junior staff [33].

### 4.4. Quality of Life, Depression, and Chronic Fatigue

The robust associations between chronic fatigue, impaired health-related quality of life, and depression confirm relationships documented in chronic fatigue literature [9,10]. These relationships demonstrate complex, bidirectional causality patterns [9,10]. Chronic fatigue directly impairs physical functioning, limiting engagement in valued activities and diminishing quality of life [9]. Reduced quality of life may exacerbate fatigue through mechanisms including deconditioning, disrupted sleep, and psychological distress [9,10].

The strong association between depression and chronic fatigue, with nearly sixfold increased risk, merits careful interpretation. Depression frequently manifests with fatigue as a core symptom [10,34]. Researchers propose that chronic fatigue and depression may share common neurobiological substrates, including hypothalamic–pituitary–adrenal axis dysregulation and altered inflammatory cytokine profiles [1,34]. The strong depression-fatigue relationship underscores the importance of comprehensive mental health screening among healthcare workers experiencing persistent fatigue [34].

The protective effects of good physical and mental quality of life (90–92% risk reduction) suggest that interventions to enhance quality of life may yield substantial benefits [9]. Interventions promoting physical activity, stress management, social connection, and work–life balance may simultaneously improve quality of life while mitigating the risk of chronic fatigue [35].

### 4.5. Obesity and Metabolic Health

The independent association between obesity and chronic fatigue (2.5-fold increased risk) aligns with evidence linking metabolic dysfunction to persistent fatigue states [20,36]. Obesity is associated with chronic low-grade systemic inflammation characterized by elevated pro-inflammatory cytokine production, implicated in fatigue pathogenesis [36,37]. Inflammatory cytokines can induce sickness behavior characterized by fatigue, reduced motivation, and cognitive impairment [37]. Obesity frequently coexists with other metabolic abnormalities, including insulin resistance and sleep-disordered breathing, each potentially contributing to fatigue [36,38].

The relationship may also involve behavioral pathways. Obesity is often associated with reduced physical activity, creating deconditioning that exacerbates fatigue [36]. Sleep quality frequently declines with obesity, particularly when obstructive sleep apnea develops [38]. These findings suggest that metabolic health optimization through weight management, physical activity promotion, and nutritional counseling may represent essential components of chronic fatigue management strategies [35,36].

### 4.6. Vitamin D and Chronic Fatigue

The absence of an independent association between vitamin D status and chronic fatigue contrasts with some studies that reported such an association [11,12]. The counterintuitive bivariate finding (lower fatigue among vitamin D-deficient individuals: 22.6% vs. 42.4%, *p* = 0.011) requires cautious interpretation. Several factors may explain this discrepancy. Tunisia’s Mediterranean location provides abundant sunlight exposure, and individuals classified as vitamin D deficient may receive sufficient sunlight to maintain adequate physiological function [19,30]. The exclusion of participants with severe chronic diseases may have removed individuals most likely to demonstrate vitamin D–fatigue associations [6,16]. Blood sampling during October–December coincides with the post-summer months, when vitamin D levels typically remain elevated [30].

The counterintuitive finding may reflect confounding by unmeasured factors, including outdoor activity levels [12]. Individuals with higher outdoor activity may have better vitamin D status and higher physical exertion, leading to fatigue. The lack of an independent association after adjustment suggests that the vitamin D–fatigue relationships observed in some studies may be mediated by psychosocial and metabolic factors [11,12]. Longitudinal studies incorporating vitamin D supplementation are needed to establish whether causal relationships exist in North African populations and to define local vitamin D deficiency thresholds [12].

### 4.7. Patient Safety Implications

The high prevalence of chronic fatigue carries profound implications for patient safety, healthcare quality, and health system performance [13,16]. Systematic reviews demonstrate that healthcare worker fatigue is associated with increased medical error rates, reduced diagnostic accuracy, impaired clinical judgment, and increased adverse patient outcomes [16,39]. The finding that mental fatigue affected 67.3% warrants emphasis, given the cognitive demands inherent in healthcare delivery. Clinical decision-making requires sustained attention, information integration, pattern recognition, and rapid problem-solving [16,39]. Mental fatigue impairs these cognitive processes, increasing vulnerability to errors [16,39].

In resource-limited settings where healthcare worker shortages result in elevated patient-to-staff ratios and extended work hours, the compounding effects of workload and fatigue may create particularly hazardous conditions [18]. Chronic fatigue among healthcare workers should be conceptualized as a critical patient-safety and health-system resilience issue [13,16]. This reframing emphasizes organizational responsibility for creating work environments that prevent fatigue development through evidence-based interventions, including workload management, shift length limitations, adequate staffing levels, and protection of recovery periods [13,39].

### 4.8. Study Limitations

Although this was, to our knowledge, the first study to measure chronic fatigue among healthcare professionals in our context, several methodological limitations warrant acknowledgment. The cross-sectional design precludes establishing temporal relationships or causal inferences. Despite random sampling, the possibility of self-selection bias due to fatigue status cannot be ruled out entirely. The predominantly female sample (89.3%) with only 22 male participants significantly limits statistical power for gender comparisons and generalizability of gender-related findings—single-center recruitment limits generalizability to other healthcare settings. The restriction to healthcare workers without severe chronic diseases may have introduced selection effects, potentially underestimating the actual fatigue burden. Reliance on self-reported questionnaires introduces potential for response bias; the use of internationally validated tools may limit this bias. The study did not collect data on some potentially confounding variables, including specific working hours, specific workload, workplace social support, dietary patterns, physical activity levels, or sleep quality. Data collection during October–December 2021, coinciding with the ongoing COVID-19 pandemic, may have influenced fatigue prevalence. The unexpected inverse relationship between vitamin D deficiency and total fatigue suggests potential unmeasured confounding.

## 5. Practical Implications

This investigation documents substantial chronic fatigue burden among healthcare professionals in a North African university hospital, with more than one-third meeting criteria for chronic fatigue and over one-tenth fulfilling chronic fatigue syndrome diagnostic criteria. General and mental fatigue dimensions demonstrated particularly high prevalence, affecting approximately two-thirds of participants and reflecting both physical exhaustion and cognitive depletion characteristic of demanding healthcare work environments. The robust independent associations among chronic fatigue, depression, obesity, and impaired health-related quality of life underscore the multifactorial etiology of fatigue in healthcare worker populations and illuminate complex interrelationships among physical health, psychological well-being, and occupational functioning.

The nearly sixfold increased chronic fatigue risk conferred by moderate-to-severe depression and the 2.5-fold risk associated with obesity, alongside the powerful protective effects of good physical and mental quality of life, reducing risk by approximately 90%, provide compelling evidence for comprehensive, multilevel interventions. Systematic screening programs for chronic fatigue, depression, and mental health symptoms among healthcare workers should be implemented through biannual assessments using validated instruments, with healthcare workers scoring above clinical thresholds receiving immediate referral to occupational health services for comprehensive evaluation and individualized intervention planning. Screening data should be systematically aggregated to enable institutional-level surveillance, early identification of high-risk units, and objective assessment of intervention effectiveness.

Comprehensive workplace wellness initiatives must address multiple interconnected health domains with adequate institutional commitment. Essential components include mental health support services providing readily accessible professional counseling; nutritional counseling and weight management programs specifically addressing obesity prevention given its demonstrated association with chronic fatigue; physical activity promotion with accessible facilities and structured programs; sleep hygiene education addressing the critical role of sleep quality; and social and work–life balance facilitation through both individual coaching and organizational policy changes including flexible scheduling and protected rest periods. 

Fundamental organizational interventions that address the root causes of excessive workload are essential. Systematic workload assessment utilizing validated tools must ensure reasonable, sustainable assignments. The exact relationship between night work and chronic fatigue should be evaluated to define the optimal shift length that balances worker health and patient safety with job demands. 

Adequate staffing levels to maintain safe patient-to-staff ratios represent the fundamental prerequisite, as chronic understaffing constitutes the root cause of excessive workload that individual-level interventions cannot adequately address. It is essential to systematically ensure protected meal and rest breaks to maintain performance during long shifts. This should be coupled with systematic fatigue risk management protocols, adapted from safety-critical industries, which must incorporate fatigue risk assessment, fitness-for-duty procedures, and evidence-based scheduling to minimize circadian disruption. 

The explicit reframing of fatigue prevention as a critical patient safety and healthcare quality improvement initiative rather than solely an employee health concern proves essential. Establishing formal fatigue risk management programs modeled on aviation safety approaches, including systematic incident reporting, examining fatigue’s contributions to adverse events, and integrating fatigue metrics into institutional quality dashboards, represents a necessary structural change. This reframing shifts responsibility from individual workers to organizational and system levels, where effective interventions must primarily operate. A multifaceted approach should be implemented, considering not only work-related factors but also social factors such as inadequate childcare, financial strain, chronic stress, and environmental factors that may cause fatigue.

Future research is needed, including longitudinal cohort studies with repeated measurements to establish temporal relationships and causal pathways. Randomized controlled intervention trials should be conducted to examine the effectiveness of multilevel workplace and mental health wellness programs, organizational modifications, and clinical treatments in reducing chronic fatigue among healthcare workers in resource-constrained settings. Multicenter investigations across diverse healthcare facilities would enhance generalizability and enable examination of organizational and social factors influencing fatigue burden. Integration of objective fatigue measures, including cognitive performance testing and actigraphy, would complement self-report instruments. Gender-balanced samples with adequate male representation are critically needed to definitively establish gender-specific patterns. Rigorous vitamin D supplementation trials with larger sample sizes and a comprehensive assessment of confounding variables are required to establish whether causal relationships exist in North African populations.

Protecting healthcare worker health through multilevel evidence-based chronic fatigue prevention strategies represents a critical investment in both workforce sustainability and health system quality, safety, and resilience. In resource-constrained environments facing healthcare worker shortages and substantial infrastructure limitations, optimizing healthcare workforce health becomes particularly compelling and cost-effective. The implementation of multilevel, evidence-based interventions should be considered an essential health system infrastructure investment, rather than optional programs subject to elimination. Failure to systematically address healthcare workers’ chronic fatigue perpetuates declining worker health, reduced workforce retention, compromised patient safety, and degradation of health system performance. 

## 6. Conclusions

This study highlighted a high prevalence of chronic fatigue and chronic fatigue syndrome among healthcare professionals without severe chronic conditions in low-resource settings. Chronic fatigue was associated with depression, obesity, and impaired quality of life. There is an urgent need for sustained and coordinated action from healthcare institutions, professional organizations, policymakers, and the global health community to prevent and protect the health of caregivers, ensure the health and quality of life, and improve healthcare security.

## Figures and Tables

**Table 1:** Characteristics of healthcare professionals participating in the study (*n* = 205), University Hospital Farhat Hached, Sousse, Tunisia, October–December 2021.

Characteristic	Category	*n* (%)
Gender	Male	22 (10.7)
	Female	183 (89.3)
Age (years)	≤40	96 (46.8)
	>40	109 (53.2)
Skin phenotype	Does not tan—very fair skin	3 (1.5)
	Tans with difficulty—very light skin	20 (9.8)
	Light skin	51 (25.4)
	Matte skin	99 (48.3)
	Dark skin	31 (15.1)
Occupation	Physician	37 (18.0)
	Nurse	90 (43.9)
	Health technician	78 (38.0)
Night work	No	148 (72.2)
	Yes	55 (27.8)
Marital status	Married	185 (90.2)
	Single	15 (7.3)
	Divorced	5 (2.4)
Hypertension	No	183 (89.3)
	Yes	22 (10.7)
Diabetes mellitus	No	192 (93.7)
	Yes	13 (6.3)
Prior COVID-19 infection	No	110 (53.7)
	Yes	95 (46.3)
COVID-19 severity *	Little symptomatic	35 (36.4)
	Not very severe	57 (59.4)
	Required oxygen	4 (4.2)
Tobacco use	No	185 (90.2)
	Yes	20 (9.8)

* Among those with prior COVID-19 infection (*n* = 95).

**Table 2:** Chronic fatigue scores (Multidimensional Fatigue Inventory, MFI-20) among healthcare professionals (*n* = 205), University Hospital Farhat Hached, Sousse, Tunisia.

MFI-20 Dimension	*n*	Mean (SD)	Median (IQR)	Min–Max	*n* (%) Meeting Threshold *
General Fatigue	205	12.40 (3.95)	12 (10–15)	4–20	136 (66.3)
Physical Fatigue	205	11.69 (3.86)	12 (9–14)	4–20	161 (78.5)
Reduced Activity	205	10.21 (4.15)	10 (7–13)	4–20	115 (56.1)
Reduced Motivation	205	10.61 (3.95)	10 (8–14)	4–18	136 (66.3)
Mental Fatigue	205	9.59 (2.57)	10 (8–11)	4–19	138 (67.3)
Total Fatigue Score	205	54.5 (14.32)	55 (43–65)	24–85	**76 (37.1)**

SD: standard deviation; IQR: interquartile range. * Threshold criteria: ≥11 for general fatigue, ≥10 for physical fatigue and reduced activity, ≥9 for reduced motivation and mental fatigue, ≥43.5 for total chronic fatigue.

**Table 3:** Chronic fatigue syndrome (myalgic encephalomyelitis (CFS/ME)) prevalence (CFS Severity Scale) among healthcare professionals (*n* = 205), University Hospital Farhat Hached, Sousse, Tunisia.

CFS Category	*n*	%
Normal status	128	62.4
Idiopathic chronic fatigue	45	22.0
CFS-like syndrome with insufficient fatigue	9	4.4
Chronic fatigue syndrome (myalgic encephalomyelitis (CFS/ME))	23	11.2

CFS Severity Scale Score: mean = 8.24 (SD = 5.14); median = 7 (IQR: 4–12); range: 0–23. CFS diagnosis criteria: Score ≥14 with moderate-to-severe fatigue.

**Table 4:** Factors associated with dimensions of chronic fatigue (Multidimensional Fatigue Inventory MFI-20) among healthcare professionals (*n* = 205), University Hospital Farhat Hached, Sousse, Tunisia.

Variable	Category	General Fatigue *n* (%)		Physical Fatigue *n* (%)		Reduced Activity *n* (%)		Reduced Motivation *n* (%)		Mental Fatigue *n* (%)		Total Fatigue *n* (%)	
		Yes	*p*	Yes	*p*	Yes	*p*	Yes	*p*	Yes	*p*	Yes	*p*
Gender	Male	7 (31.8)	**<0.001**	15 (68.2)	0.269	11 (50.0)	0.086	7 (31.8)	**0.015**	14 (63.6)	0.697	3 (13.6)	**0.016**
	Female	129 (70.5)		146 (79.8)		125 (68.3)		108 (59.0)		124 (67.8)		73 (39.9)	
Age (years)	≤40	71 (74.0)	**0.030**	74 (77.1)	0.634	61 (63.5)	**0.044**	67 (69.8)	0.327	70 (72.9)	0.109	37 (38.5)	0.683
	>40	65 (59.6)		87 (79.8)		54 (49.5)		69 (63.3)		68 (62.4)		39 (35.8)	
Profession	Doctor	27 (73.0)	0.346	30 (81.1)	0.677	24 (64.9)	0.235	27 (73.0)	0.346	24 (64.9)	0.725	13 (35.1)	0.787
	Nurses	109 (64.9)		131 (78.0)		91 (54.2)		109 (64.9)		114 (67.9)		63 (37.5)	
Married	No	13 (65.0)	0.894	11 (55.0)	**0.007**	9 (45.0)	0.292	14 (70.0)	0.715	9 (45.0)	0.250	6 (30.0)	0.491
	Yes	123 (66.5)		150 (81.1)		106 (57.3)		122 (65.9)		129 (69.7)	70 (37.8)		
Night work	No	101 (68.2)	0.353	113 (76.4)	0.219	87 (58.8)	0.212	100 (67.6)	0.549	97 (65.5)	0.382	52 (35.1)	0.355
	Yes	35 (61.4)		48 (84.2)		28 (49.1)		36 (63.2)		41 (71.9)		24 (42.1)	
Tobacco use	No	126 (68.1)	0.104	144 (77.8)	0.576	106 (57.3)	0.292	121 (65.4)	0.388	123 (66.5)	0.441	71 (38.4)	0.239
	Yes	10 (50.0)		17 (85.0)		9 (45.0)		15 (75.0)		15 (75.0)		5 (25.0)	
COVID-19	No	69 (62.7)	0.239	87 (79.1)	0.835	55 (50.0)	0.058	68 (61.8)	0.140	74 (67.3)	0.988	37 (33.6)	0.273
	Yes	67 (70.5)		74 (77.9)		60 (63.2)		68 (71.6)		64 (67.4)		39 (41.1)	
Sun exposure	No	82 (71.9)	0.058	89 (78.1)	0.856	67 (58.8)	0.388	81 (71.1)	0.110	78 (68.4)	0.706	48 (42.1)	0.095
	Yes	54 (59.3)		72 (79.1)		48 (52.7)		55 (60.4)		60 (65.9)		28 (30.8)	
Vitamin D	<20	33 (62.3)	0.445	39 (73.6)	0.289	32 (60.4)	0.436	35 (66.0)	0.861	32 (60.4)	0.135	12 (22.6)	**0.011**
	>20	98 (68.1)		116 (80.6)		78 (54.2)		97 (67.4)		103 (71.5)		61 (42.4)	
Sun exposure during vacation	Head only	75 (76.5)	**0.008**	78 (79.6)	0.325	61 (62.2)	**0.044**	70 (71.4)	0.334	71 (72.4)	0.251	44 (44.9)	**0.026**
	Head, arms, legs	47 (59.5)		64 (81.0)		44 (55.7)		49 (62.0)		51 (64.6)		27 (34.2)	
	Body/swimsuit	14 (50.0)		19 (67.9)		10 (35.7)		17 (60.7)		16 (57.1)		5 (17.9)	
HRQoL (PCS)	Poor	112 (76.7)	**<0.001**	125 (85.6)	**<0.001**	97 (66.4)	**<0.001**	114 (78.1)	**<0.001**	110 (75.3)	**<0.001**	72 (49.3)	**<0.001**
	Good	24 (40.7)		36 (61.0)		18 (30.5)		22 (37.3)		28 (47.5)		4 (6.8)	
HRQoL (MCS)	Poor	120 (72.7)	**<0.001**	136 (82.4)	**0.006**	104 (63.0)	**<0.001**	120 (72.7)	**<0.001**	121 (73.3)	**<0.001**	73 (44.2)	**<0.001**
	Good	16 (40.0)		25 (62.5)		11 (27.5)		16 (40.0)		17 (42.5)		3 (7.5)	
CFS	No	113 (62.1)	**<0.001**	138 (75.8)	**0.005**	93 (51.1)	**<0.001**	113 (62.1)	**<0.001**	121 (66.5)	0.474	55 (30.2)	**<0.001**
	Yes	23 (100)		23 (100)		22 (95.7)		23 (100)		17 (73.9)		21 (91.3)	
Depression	No	99 (60.4)	**<0.001**	121 (73.8)	**0.001**	77 (47.0)	**<0.001**	99 (60.4)	**<0.001**	103 (62.8)	**0.006**	43 (26.2)	**<0.001**
	M/S *	37 (90.2)		40 (97.6)		38 (92.7)		37 (90.2)		35 (85.4)		33 (80.5)	
Obesity	Normal/overweight	103 (75.2)	0.210	52 (72.2)	0.126	72 (52.6)	0.383	86 (62.8)	0.052	90 (65.7)	0.383	42 (30.7)	**0.002**
	Obesity	55 (85.9)		55 (85.9)		41 (64.1)		49 (76.6)		46 (71.9)		34 (53.1)	

* M/S = moderate-to-severe depression; HRQoL: health-related quality of life; PCS: Physical Component Summary; MCS: Mental Component Summary; CFS: chronic fatigue syndrome. Bold *p*-values indicate statistical significance (*p* < 0.05). Chi-square (or Fisher’s exact, where appropriate) test was used to compare percentages between groups.

**Table 5:** Multivariable logistic regression analysis of factors independently associated with chronic fatigue (Multidimensional Fatigue Inventory MFI-20) among healthcare professionals (*n* = 205), University Hospital Farhat Hached, Sousse, Tunisia.

Factor	Adjusted OR	95% CI	*p*-Value
Good physical health-related quality of life (PCS-12 ≥ 50)	0.08	0.03–0.25	<0.001
Good mental health-related quality of life (MCS-12 ≥ 50)	0.10	0.03–0.39	<0.001
Moderate-to-severe depression (PHQ-9 ≥ 10)	5.84	2.32–14.71	<0.001
Obesity (BMI ≥ 30 kg/m^2^)	2.50	1.15–5.46	0.021

Nagelkerke R^2^ = 48.3%. Notes: OR: odds ratio; CI: confidence interval; PCS-12: Physical Component Summary of SF-12; MCS-12: Mental Component Summary of SF-12; PHQ-9: Patient Health Questionnaire-9; BMI: body mass index model adjusted for age, gender, occupation, marital status, and vitamin D level.

## Data Availability

The data supporting the findings of this study are not publicly available due to privacy and ethical restrictions, as they contain sensitive information about study participants. The datasets generated during the current study are available from the corresponding author upon reasonable request.
